# Potential therapeutic effects of ibudilast and retinoic acid against cuprizone-induced behavioral and biochemical changes in mouse brain

**DOI:** 10.3389/fnmol.2025.1567226

**Published:** 2025-05-20

**Authors:** Kholoud A. Alyami, Gadah A. Alshahrany, Kholoud M. Al-Otaibi, Mohammad Z. Alam, Badrah S. Alghamdi, Hadeil M. Alsufiani, Nouf O. Alshareef, Hanna M. Alhoraibi, Sahar A. Alkhodair, Ulfat M. Omar

**Affiliations:** ^1^Department of Biochemistry, Faculty of Science, King Abdulaziz University, Jeddah, Saudi Arabia; ^2^Department of Chemistry, Faculty of Science, Albaha University, Albaha, Saudi Arabia; ^3^Department of Medical Laboratory Sciences, Faculty of Applied Medical Sciences, King Abdulaziz University, Jeddah, Saudi Arabia; ^4^Neuroscience and Geroscience Research Unit, King Fahd Medical Research Center, King Abdulaziz University, Jeddah, Saudi Arabia; ^5^Department of Physiology, Neuroscience Unit, Faculty of Medicine, King Abdulaziz University, Jeddah, Saudi Arabia; ^6^Experimental Biochemistry Unit, King Fahd Medical Research Center, King Abdulaziz University, Jeddah, Saudi Arabia; ^7^Princess Dr. Najla Bint Saud Al-Saud Center for Excellence Research in Biotechnology, King Abdulaziz University, Jeddah, Saudi Arabia

**Keywords:** multiple sclerosis, ibudilast, retinoic acid, vitamin A, cuprizone

## Abstract

Ibudilast (IBD) is a new drug that has been released as treatment for multiple sclerosis (MS). Retinoic acid (RA), a metabolite of vitamin A, is known for its pro-regenerative and anti-inflammatory properties, therefore, it has been suggested as a supplementary treatment for MS. The objective of this study is to investigate the therapeutic effects of RA and IBD against cuprizone (CPZ) induced mouse models. Seventy-two Swiss Albino male Mice (SWR/J) were divided into two main groups control (*n* = 18); normal chow and CPZ (*n* = 54); 0.25% of CPZ mixed into chow at demyelination stage (first 5 weeks). The following 4 weeks included two stages of remyelination: early remyelination (2 weeks after CPZ discontinuation) and late remyelination (week 9). In the early stage of remyelination, the CPZ group was divided into four subgroups beside daily treatment intraperitoneal injections CPZ (+ve control- no treatment), RA (20 mg/kg), IBD (10 mg/kg), and RA + IBD, with (*n* = 12/group), while the control group had 12 mice. At the end of each stage 6 mice/ group were sacrificed. Mice response to different treatments was assessed using several locomotor and cognitive behavior tests including open field test, rotarod test, grip strength test, novel object recognition test (NORT) and Y-maze test. The expression levels of several genes MS associated genes Tumer Necrosis Factor- Alpha (TNF- *α*), Cyclooxygenase-2 (COX-2), Nerve Growth Factor (NGF), Signal transducer and activator of transcription 3 (STAT-3) and Nuclear factor kappa-light-chain-enhancer of activated b-cell (NFKB-P105) in the brain of mice were measured using quantitative Reverse Transcription Polymerase Chain Reaction (qRT-PCR) analysis. The results demonstrated that RA supplementation helped in alleviating the symptoms of MS induced mice with or without using IBD treatment. This was indicated as an improvement in locomotor activity, motor coordination and muscular strength as well as improving the cognition and memory functions. The mRNA expression pattern of various MS associated genes indicated that the treatments effectively mitigated the detrimental effects of CPZ in mouse brain. The findings of this study indicate that RA supplements could be effectively unitized as adjuvant therapy alongside with IBD for MS treatment.

## 1 Introduction

Neurodegenerative diseases (NDs) are a variety of heterogeneous disorders which are characterized by loss of neurons associated with protein deposition leading to progressive destruction of central nervous system (CNS) structure and function (Garofalo et al., 2020; Wilson et al., 2023). Multiple sclerosis (MS) is one of the most popular neurodegenerative diseases that typically occurs during the most productive period of life, between 20 and 40 years (Walton et al., 2020; Yamout and Alroughani, 2018; Sandal Kilic et al., 2025). According to the MS atlas, the disease affects over 2.8 million individuals globally, with a higher prevalence observed in females compared to males (Voskuhl et al., 2020; Gold et al., 2019).

The formation of plaques in the white and gray matter of the brain as well as throughout the CNS is a hallmark of MS. These plaques are characterized by varying levels of neuroinflammation, gliosis, neurodegeneration, and axonal loss (Popescu et al., 2013; Kreiter et al., 2024). The process begins with the release of autoantigens from the CNS into peripheral blood, where they are recognized by dendritic cells. This recognition activates T-cells in the peripheral circulation, which then interact with specific adhesion molecules on brain microvascular endothelial cells (BMEC) and migrate through the blood–brain barrier (BBB) into the CNS (Baecher-Allan et al., 2018; Gold and Wolinsky, 2011). Once the T-cell within CNS is re-activated by local antigen presenting cells (APCs) such as macrophages, it leads to the production of pro-inflammatory cytokines such as Interferon gamma (INF-*γ*), Tumor Necrosis Factor Alpha (TNF- *α*), Interleukin-6 (IL-6) and Interleukin-17 (IL-17). These cytokines chronically activate resident cells in CNS such as astrocytes and microglia cells (Kamm et al., 2014; Loma and Heyman, 2011). Once antibodies bind to the myelin sheath, macrophages initiate phagocytosis of the myelin marked by these antibodies (Cosentino and Marino, 2013). As a result of myelin degeneration around axons plaques were formed. Clinically, these plaques translated into initial relapses that evolve over time into progressive severity and disability in MS patients (Christogianni et al., 2018; Grzegorski and Losy, 2017).

The exact etiology of MS remains undetermined; however, both environmental and genetic factors contribute to an increased risk of developing the disease (Leray et al., 2016; Shi et al., 2024). To date, the MS disease remains incurable; however, the US Food and Drug Administration (FDA) and European Medicines Agency (EMA) have approved fifteen disease modifying therapies (Hauser and Cree, 2020; Reich et al., 2018). Ibudilast (IBD), is a novel drug classified as a pyrazolo pyridine, an organic compound known for its therapeutic potential. It is a nonselective inhibitor of cyclic nucleotide phosphodiesterase (PDE) that can cross blood–brain barrier, inhibits glial activation and produces macrophage migration inhibitory factor (Ray et al., 2014; Rolan et al., 2009). *In vitro* study revealed that IBD is also a powerful anti-inflammatory agent with neuro-protective effects (Mizuno et al., 2004). Furthermore, IBD enhances the levels of cyclic adenosine monophosphate (cAMP) and cyclic guanosine monophosphate (cGMP), reduces the pro-inflammatory cytokines (IL-1β, IL-6, and TNF-*α*), reactive oxygen species (ROS) and nitric oxide (NO) and improves the synthesis of anti-inflammatory substances such as IL-10, Nerve growth factor (NGF), neurotrophin-4, and glial cell-line derived neurotrophic factor (Goodman et al., 2016; Sharifian et al., 2023; Kolahdouzan et al., 2019). Many studies found that IBD offers therapeutic benefits in both clinical trials and pre-clinical studies using animal models of MS such as experimental autoimmune encephalomyelitis (EAE) (Angelopoulou et al., 2022).

Retinoic Acid (RA), also known as vitamin A, is a compound that has been extensively studied for its effectiveness in immunomodulation. It reduces pro-inflammatory cytokines such as IL-17, TNF-α, IL-1β, and IL-12, while enhancing anti-inflammatory cytokines like IL-10 (Khosravi-Largani et al., 2018; Wang et al., 2007). Based on the biological activities of RA, it is considered as a promising candidate for modulating immune responses and facilitating axonal regeneration in MS (Ruschil et al., 2021). RA has been shown to exert neuroprotective effects in astrocyte through bind to RXR receptor which regulates the expression of several anti-inflammatory genes (Goodman et al., 2016). In EAE model, RA demonstrated synergistic effects with atorvastatin by reducing pro-inflammatory cytokines and enhancing anti-inflammatory cytokines (Abtahi Froushani et al., 2014). However, none of these studies have reported the effect of IBD and RA administration on CPZ animal models of MS. Therefore, this study aimed to explore the potential therapeutic effect of IBD and RA administration individually and in combination on CPZ-induced toxicity in mouse brain.

The Cuprizone (CPZ) is a copper-chelating agent that induces neurotoxicity in rodent CNS by provoking oligodendrocyte apoptosis, along with microgliosis and astrogliosis in corpus callosum (CC) (Gudi et al., 2009; Mooshekhian et al., 2022; Patergnani et al., 2017). This model is commonly used to study CNS demyelination and spontaneous remyelination following CPZ withdrawal from the diet (Gharagozloo et al., 2022; Samtani et al., 2023). Research has demonstrated that a few days of CPZ intoxication disrupts the homeostasis of essential ions, such as sodium, manganese, zinc, iron, and copper, in various regions of the body (Moldovan et al., 2015; Varga et al., 2018) As a result, both endoplasmic reticulum (ER) and mitochondrial functions are compromised in the brain and liver of mice (Acs et al., 2013; Solti et al., 2015). The formation of megamitochondria plays a significant role in the progression of nervous system degeneration, which is a key factor in neurodegenerative diseases. This leads to the inhibition of metalloenzymes, such as monoamine oxidase, ceruloplasmin, cytochrome oxidase, and superoxide dismutase, which contain copper as a cofactor (Ruiz et al., 2021). As a consequence, adenosine triphosphate (ATP) production declines, and antioxidant enzyme levels, which are responsible for neutralizing harmful reactive oxygen species (ROS) and reactive nitrogen species (RNS), are reduced (Faizi et al., 2016; Vega-Riquer et al., 2019). As a result, concentration of ROS and RNS increases, which ultimately disrupts ER function (Di Meo et al., 2016). This disturbance causes a decrease in mRNA levels, the accumulation of misfolded proteins, and triggers the unfolded protein response (UPR). This response leads to a massive influx of calcium into the cytosol, ultimately causing stress in oligodendrocytes (OLGs) (Malhotra and Kaufman, 2007).

To complete OLG apoptosis in the CPZ model, the activation of innate immune cells such as microglia, macrophages, and astrocytes occurs, facilitating the phagocytosis of myelin. These events are accompanied by the release of inflammatory mediators like Nitric Oxide (NO), Interleukin-1 beta (IL-1β), TNF-*α*, IFNγ, and IL-6. The apoptosis of OLGs leads to demyelination in the white matter areas, including the corpus callosum (CC), internal capsule, thalamus, anterior commissure, and cerebellar peduncles (Vega-Riquer et al., 2019). After CPZ withdrawal, myelin protein’s re-expression and remyelination occurs.

As consequence of neuroinflammation induced by CPZ behavioral changes such as weakness, lethargy, and weight loss were initially assessed qualitatively through visual observation (Petronilli and Zoratti, 1990). Since then, several studies have documented behavioral alterations caused by CPZ intoxication, including motor impairments, muscle weakness, and cognitive deficits (Kondo et al., 2016; Elbaz et al., 2018).

To the best of our knowledge, no previous study has investigated the therapeutic effect of combining RA and IBD in the CPZ mouse model. Therefore, the aim of this study is to investigate the potential therapeutic effect of IBD and RA against MS in CPZ mouse model on promoting remyelination and improving motor behavioral deficits during both early and late stages of remyelination in the CPZ-induced demyelination model, as well as exploring the potential benefits of their combination.

## 2 Materials and methods

### 2.1 Experimental animals

A total of 72 SWR/J male mice aged 6–7 week, weighing 18 to 25 g were obtained from the animal house unit at King Fahd Medical Research Center (KFMRC), King Abdulaziz University (KAU), Jeddah, Saudi Arabia. Mice were housed as five mice per a standard cage (33 × 15 × 13 cm) and the cage was cleaned twice a week. All mice were maintained under standard environment conditions (12:12 h light–dark cycle, at room temperature 23.0 ± 1.0°C and humidity of 60.0 ± 5.0% with ad libitum). All animal experiments were performed according to the guidelines of biomedical ethics committee (Reference No. 617–20) at KAU following the rules and regulations of the Animal Care and Use Committee at KFMRC, which complied with the “System of Ethics of Research on Living Creatures” guidelines prepared by King Abdulaziz City for Science and Technology. The protocol of this current research is approved by the biomedical ethics research committee at KAU with the approval No. ACUC-20-10-24.

### 2.2 Preparation of the drugs

#### 2.2.1 Cuprizone

CPZ (C9012-25G) was purchased from Thermo-Fisher Scientific (Waltham, MA, USA). To induce demyelination in mice, CPZ was administrated for 5 weeks at a concentration of 0.25%. This was prepared by mixing approximately 0.25 g of CPZ with 100 g of ground standard rodent chow and 100 mL of water, added gradually. Rodent chow mixed with CPZ was prepared weekly and replaced daily (Zhan et al., 2020; Templeton et al., 2019).

#### 2.2.2 Retinoic acid

RA (R2625-1G) was obtained from Sigma- Aldrich (St. Louis, Missouri, United States). RA was freshly prepared by dissolving it into normal saline containing 5% DMSO. Each mouse received a daily dose of 20 mg/kg of RA administered via intraperitoneal injection (IP) in a volume of 0.2 mL during the noon time (between 12:00 PM and 2:00 PM). This dosage was selected based on previous research (Arrieta et al., 2011). The weights of mice were measured weekly, and the dosages of RA were adjusted accordingly.

#### 2.2.3 Ibudilast

IBD (KC-404-2G) was purchased from Adooq Bioscience® Company (Barranca Pkwy, Irvine, CA). IBD was freshly prepared by dissolving it in normal saline containing 5% DMSO (Kagitani-Shimono et al., 2005). Each mouse received a daily dose of 10 mg/kg of IBD administered via intraperitoneal injection (IP) in a volume of 0.2 mL during the noon time (between 12:00 PM and 2:00 PM). This dosage was selected based on previous research (Kagitani-Shimono et al., 2005). The weights of mice were measured weekly, and the dosages of IBD were adjusted accordingly.

### 2.3 Experimental design

The total duration of this experiment was 9 weeks divided into three stages: demyelination (weeks 1–5), early remyelination (weeks 6–7) and late remyelination (weeks 8–9). During the demyelination stage (weeks 1–5), 72 mice were divided into two primary groups: control group (18 mice) which was given a regular rodent chow diet and CPZ group (54 mice) which received a rodent chow diet containing 0.25% CPZ. After 5 weeks, 6 mice from these two main groups were sacrificed to perform the measurements.

**Figure 1:**
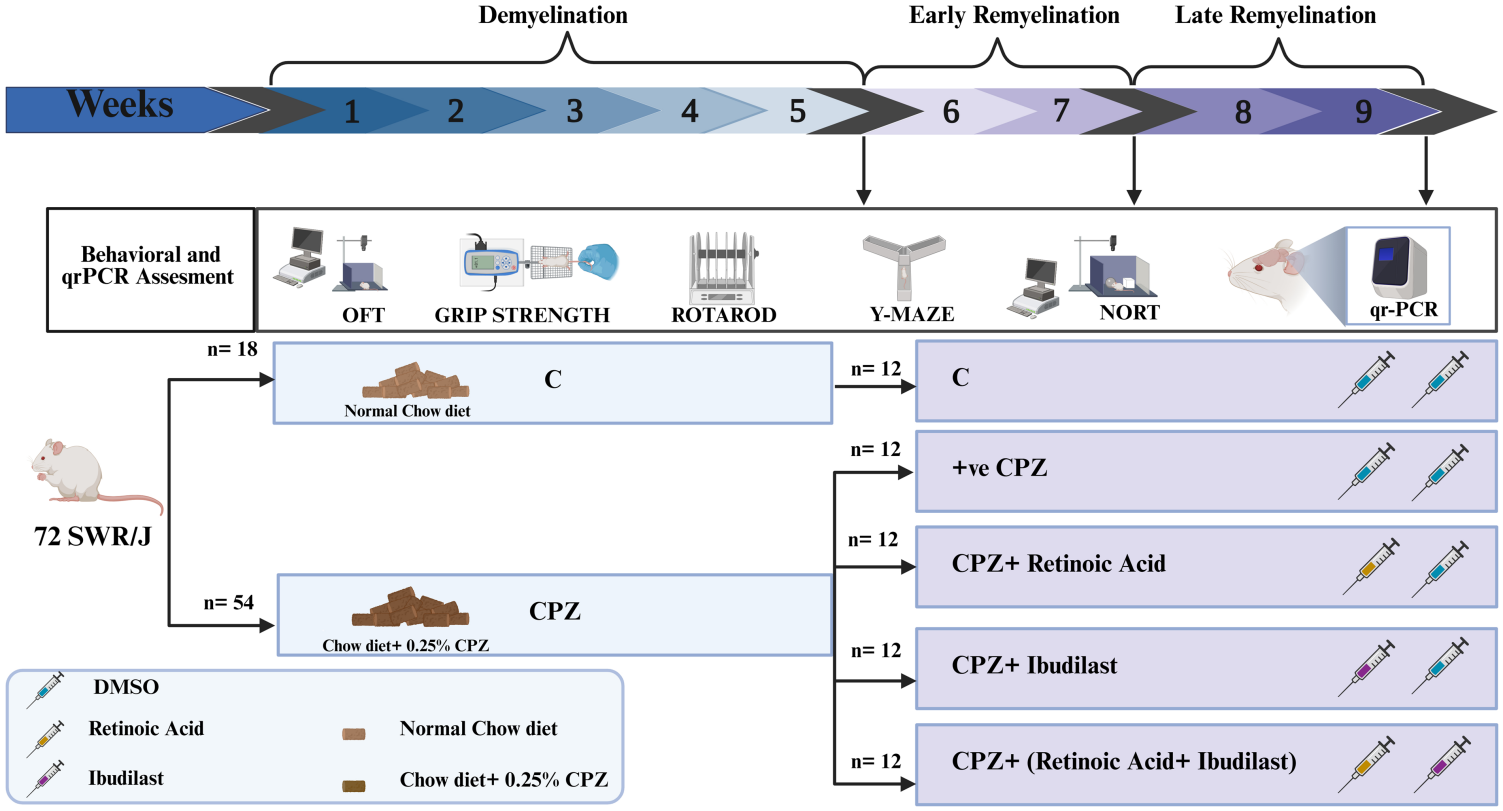
Experimental design. The experimental procedure and the timeline of inducing multiple sclerosis (MS) through CPZ (demyelination) and the spontaneous remyelination stages (early and late). After MS induction by CPZ, mice were switched to normal chow diet to induce remyelination and divided into four groups: Control (CPZ mice with no therapeutic treatment), RA (CPZ mice treated with RA), IBD (CPZ mice treated with IBD) and RA + IBD (CPZ mice treated with combination of RA and IBD treatment).

The subsequent 4 weeks included two stages of remyelination: early remyelination [occurring 2 weeks after CPZ withdrawal (weeks 6–7)] and late remyelination (occurring at the end of week nine). During the early remyelination stage, the CPZ group was divided into four subgroups based on the intraperitoneal treatments they received: CPZ group (5% DMSO), RA group (10 mg/kg-daily), IBD group (20 mg/kg-daily) and RA + IBD group (10 mg/kg + 20 mg/kg – sequentially, daily), with ensuring that the average weight in each group was equal, in addition to the primary control group (5%DMSO). Each group consisted of 12 mice. Around 30 mice (6/group) were euthanized by the end of the early remyelination stage for measurements. Finally, at the late stage of remyelination, the remaining 6 mice in each group were sacrificed by the end of week 9 for measurements (Figure 1). All mice in each stage were used to assess the behavior.

### 2.4 Behavioral tests

#### 2.4.1 Open field test

The open-field test (OFT) was conducted to evaluate the exploratory and locomotor activity of mice (Gould et al., 2009; Seibenhener and Wooten, 2015). The open field apparatus consisted of a square chamber (45 × 45 cm^2^) enclosed by walls; height 34 cm high. During the testing phase, each mouse was positioned in the corner of the chamber and allowed to acclimate for 3 min. The movement of the mouse was tracked and recorded using a video camera mounted above the arena (Alghamdi and Abo Taleb, 2020). The EithoVision tracking system (Noldus Information Technology, Wageningen, the Netherlands) was used to quantitively assess the total distance moved (TDM) in centimeters and the velocity (cm/s) during the observation period.

#### 2.4.2 Rotarod test

Motor coordination and balance in mice were evaluated using a rotarod apparatus (BR1001, B.S Technolab INC., Seoul, South Korea). Mice were placed on a rotating drum with accelerating speed of 4 to 40 rpm over a duration of 300 s, and the latency to fall was recorded (Shoji et al., 2023; Maity-Kumar et al., 2022). Each mouse underwent three trials within a 60-min rest period in-between each trial (Hattori et al., 2012). Falls occurring within the first 5 s were not considered due to poor positioning, and a brief rest time was allowed before retesting.

#### 2.4.3 Grip strength test

The Neuromuscular function of mice was assessed using Grip Strength Meter (GSM) (Columbus Instruments, Columbus, OHIO 43204, USA). Each mouse was positioned on a grid and allowed to grasp the grids with its forelimbs, the grid was then slowly pulled backward until the mouse release its grip. The maximum peak force exerted by the mouse was measured in Newtons (N). Each mouse underwent three trials, and the average force (g) was calculated considering body weight (force/body weight) for each individual mouse (Han et al., 2020).

#### 2.4.4 Novel object recognition test

Rodent memory and learning were evaluated using the novel object recognition test (NORT) (Labban et al., 2021). This test was performed over 2 days: Habituation Day and Test Day. On habituation day, each mouse was placed in an empty arena and allowed to move freely for 10 min. Test day (which occurs after 24 h) consists of two phases: familiarization and novel phases. During familiarization phase, two identical “called familiar” objects (F1, F2) were placed in opposing quadrants of the arena while a mouse is placed in the center, allowing it to investigate both objects for 3 min. The test phase started 10 min after the familiarization phase, where one of the objects was replaced with a novel object (N) in the same location. The mouse was then returned to the center of the arena for another 3 min to explore both the familiar and novel objects. After each test, the arena was washed with 10% ethanol to eliminate any olfactory cues. The EthoVision XT8A video tracking device automatically recorded the frequency of sniffing as well as the time spent exploring each object. The percentage of sniffing frequency was calculated using equation (Equation 1) and the ability of memory was assessed using the discrimination index (DI) which was calculated using equation (Equation 2):


(1) $$\begin{array}{l} \text{Frequency of sniffing}\;(\%)=\\ \frac{\text{novel or familiar object frequency of sniffing}}{\text{total frequency of sniffing of the}\;\mathrm{two}\;\text{objects}}\times 100 \end{array}$$

(2) $$\begin{array}{l} \text{Discrimination index}\;(\mathrm{DI})=\\ \frac{\begin{array}{l} \text{Novel Object Exploration Time}–\\ \text{Familiar Object Exploration Time} \end{array}}{\text{Total Exploration Time}}\times 100 \end{array}$$

#### 2.4.5 Y-maze test

The Y-maze spontaneous alternation test was used to assess short-term spatial recognition memory in mice (Kraeuter et al., 2019). The Y-maze consists of three identical arms labeled A, B, and C that are arranged at 120-degree angles, with walls measuring 10 cm wide and 40 cm high. Each mouse was placed within arm (A), facing wall and allowed to freely move through the maze for 8 min (Prieur and Jadavji, 2019). The number of entries into the arms was manually recorded when the four limbs crossed the entry point excluding the initial entry into arm (A). Alternation was calculated when the mouse visited three different arms consecutively (CBA, ABC, BCA). The percentage of spontaneous alternation was calculated using the equation (Equation 3) (Adilijiang et al., 2015).


(3) $$\;\text{Alternation}\;(\%)=\frac{\#\text{alternatio}}{\text{total}\;\mathrm{arm}\;\text{visit}-2}\times 100$$

### 2.5 Samples collection

By the end of week five and week nine, a random selection of half of the mice in each group was chosen for euthanasia via 5% of isoflurane anesthesia and subsequent decapitation. Plexiglass chamber containing Isoflurane 5% (Baxter healthcare, Deerfield, Illinois, United States) was used to anesthetize the mice until they were fully anesthetized, as confirmed by the absence of a tail pinch reflex and the lack of spontaneous movement and then decapitated by placed them on the guillotine, and their heads chopped off. The entire brain of each mouse was then obtained through dissection. The collected brains were kept in RNA-later solution for future analysis and stored at −80°C.

### 2.6 RNA isolation and quantitative real-time PCR (qRT-PCR)

Total RNA was extracted from mouse brain tissue using Trizol-chloroform method (Sharma et al., 2023). Complementary DNA (cDNA) was synthesized from RNA using SuperScript™ IV First-Strand Synthesis System (Cat No.: 18091050, Invitrogen™) according to the manufacturer’s instructions. The expression levels of mRNA of five genes (TNF-*α*, COX-2, NGF, STAT-3 and NFKB-P105) were quantified using qRT-PCR using PowerUp™ SYBR Green PCR Master Mix (Applied Biosystems). Briefly, 100 ng of cDNA was added to 5 mL of SYBR Green PCR Master Mix and 1 mL of each primer (forward and reverse primers). The concentration of each primer was 10 pmol. The qPCR reaction conditions were: 50°C for 2 min, followed by one cycle of activation of Dual-Lock™ DNA polymerase at 95°C for 2 min, followed by 40 cycles of 95°C for 15 s and anneal/extend at 60°C for 1 min (if primer Tm ≥ 60°C) and a final extension at 72°C for 60 s. For primers having Tm less than 60°C, the annealing temperature was determined for each primer set (52–59°C). The qPCR was performed using StepOne™ Real-Time PCR System (Applied Biosystems). Glyceraldehyde-3-phosphate dehydrogenase (GAPDH) was used as a reference gene. The fold change was calculated using Livak method (2^^ΔΔct^) (Livak and Schmittgen, 2001; Schmittgen et al., 2000). The primers sequences used for qPCR are provided in Supplementary Table S1.

### 2.7 Statistical analysis

All data are expressed as a mean standard error of the mean (SEM) and were statistically analyzed using GraphPad Prism 10.1.0. Unpaired *T*-test analysis was used to compare relative expression fold and behavioral parameters during the demyelination stages. One-way analysis of variance (ANOVA) followed by *post hoc* Tukey’s test was used to compare differences between groups in relative expression folds and behavioral tests. A two-way analysis of variance (ANOVA) followed by Šídák’s post hoc test was used to analyze NORT data across all stages. Differences between groups were considered statistically significant when *p* < 0.05.

## 3 Results

### 3.1 Effect of IBD and RA and their combination on motor function of CPZ-induced MS mice

#### 3.1.1 Effects of IBD and RA on locomotor activity

**Figure 2:**
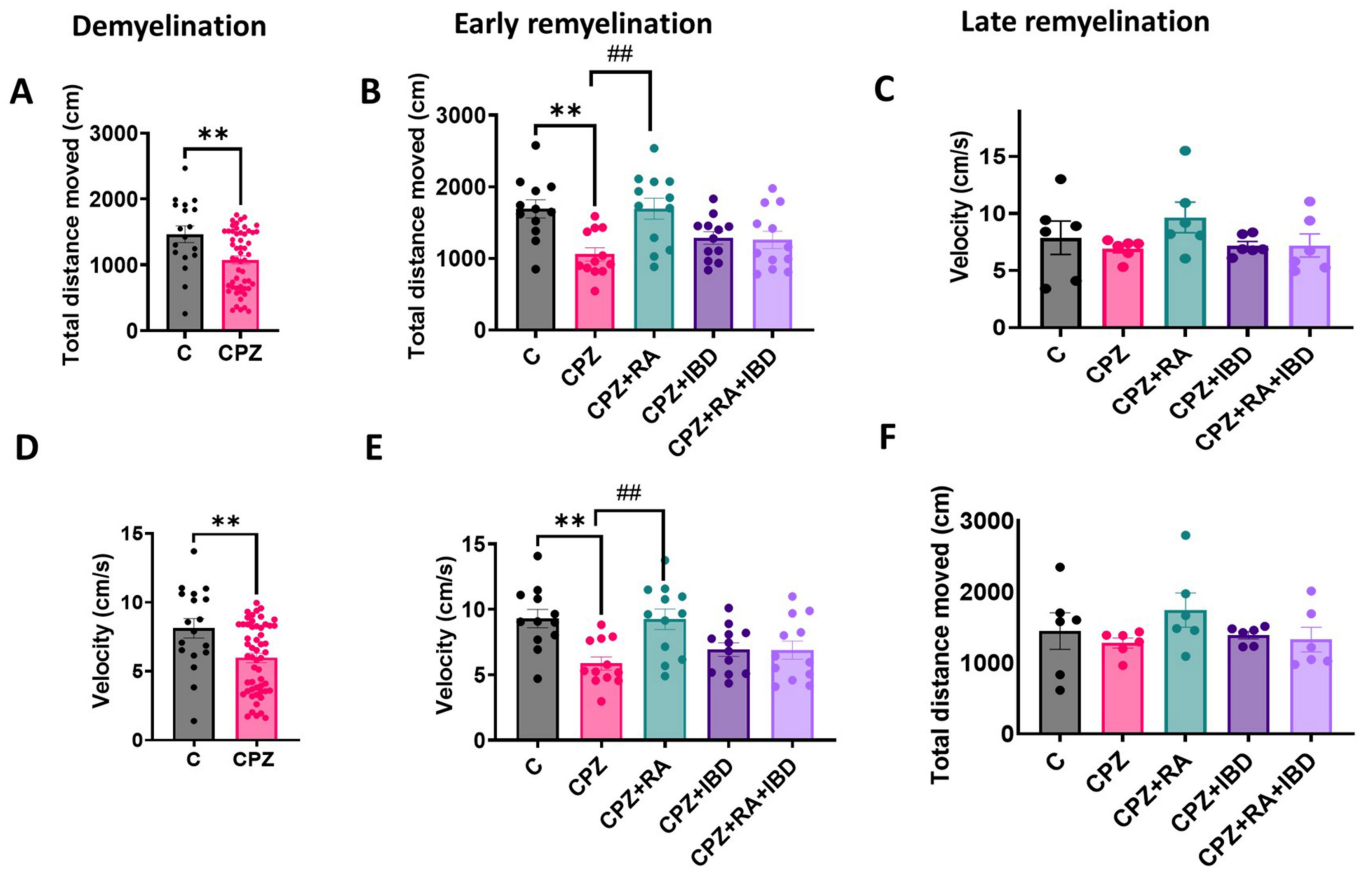
Locomotor activity of CPZ-induced MS mice. **(A–C)** Total distance movement during demyelination and (early and late) remyelination stage, respectively. **(D–F)** Velocity of movement during demyelination and (early and late) remyelination stage, respectively. At demyelination *n* = 18/C group and *n* = 54/CPZ group, early remyelination *n* = 12/group, late remyelination *n* = 6/group. * Indicates a significant difference vs. the control group; # indicates a significant difference vs. CPZ group. **p < 0.01, and ^##^*p* < 0.01.

The therapeutic effects on IBD and RA and their combination on the locomotor activity of CPZ-induced MS mice was assessed by measuring the total distance moved TDM and velocity in an open field. At demyelination stage, the CPZ group showed significant decrease in TDM, and velocity compared to C group (*p* = 0.0036 and *p* = 0.0037 respectively) (Figures 2A,D). At the end of early remyelination stage, the comparison between groups revealed significant differences in TDM [*F*_(4, 55)_ = 5.871, *p* = 0.0005] and in velocity [*F*_(4, 55)_ = 5.640, *p* = 0.0007]. The CPZ group showed a significant decrease in TDM, and velocity compared to C group (*p* = 0.0029, and *p* = 0.0040 respectively). On the other hand, the comparison showed a significant increase in TDM and velocity in RA group compared to CPZ group (*p* = 0.0028, and *p* = 0.0048 respectively). Conversely, there was a non-significant difference shown between CPZ and mice treated with IBD (*p* = 0.6419) and RA + IBD (*p* = 0.7422) (Figures 2B,E).

At the end of the late remyelination stage, the comparison between groups revealed significant differences in TDM [*F*_(4, 25)_ = 1.034, *p* = 0.4095] and in velocity [*F*_(4, 25)_ = 1.195, *p* = 0.3374] (Figures 2E,F). However, The CPZ group showed a non-significant difference in TDM, and velocity compared to C group (*p* = 0.9623, and *p* = 0.9605 respectively). Although there was a non-significant difference in TDM and velocity between CPZ and mice treated with (*p* = 0.3788, and *p* = 0.3396 respectively), IBD (*p* = 0.9920, and *p* = 0.9996 respectively), and RA + IBD (*p* = 0.9996, and *p* = 0.9996 respectively) (Figures 2C,F).

#### 3.1.2 Effects of IBD and RA and their combination on motor coordination

**Figure 3:**
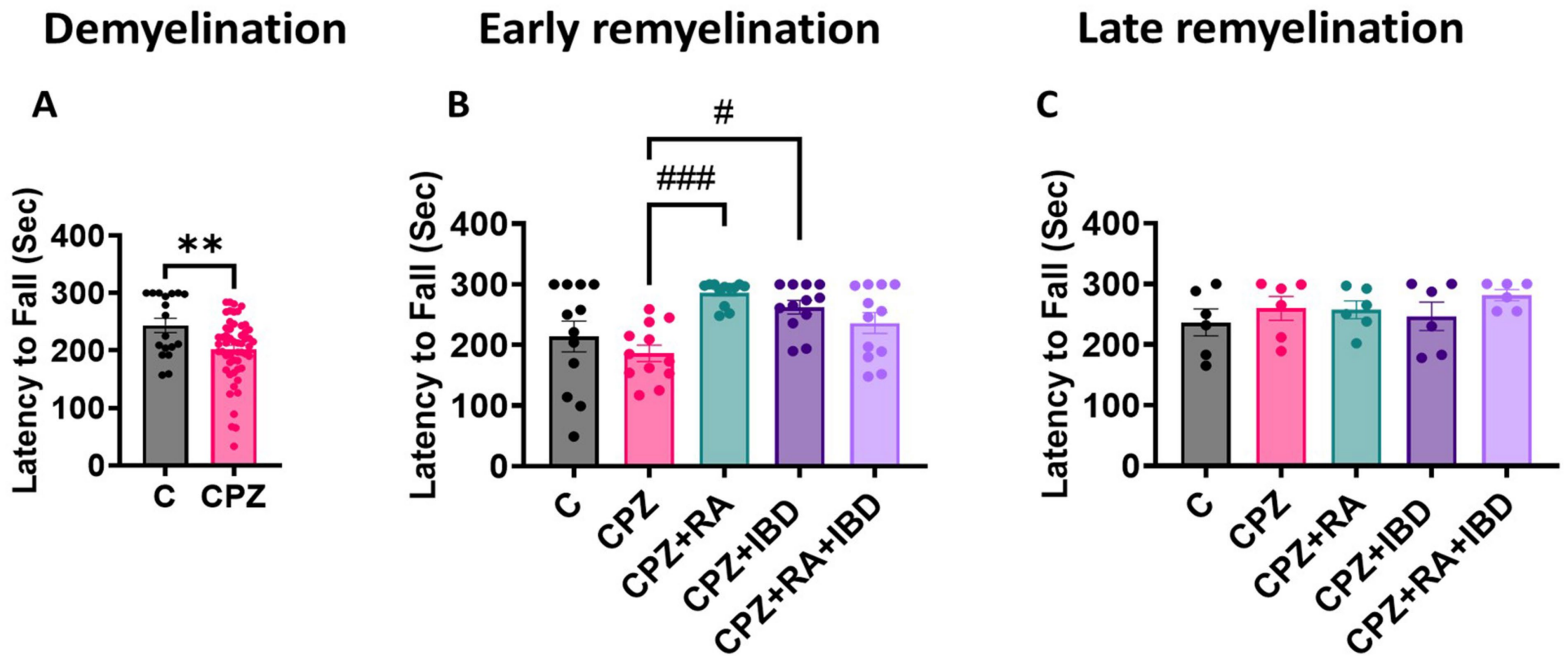
Motor coordination in CPZ-induced MS mice. **(A)** Latency to fall at demyelination stage. **(B)** Latency to fall at early remyelination stage. **(C)** Latency to fall at late remyelination stage. At demyelination *n* = 18/C group and *n* = 54/CPZ group, early remyelination *n* = 12/group, late remyelination *n* = 6/group. * Indicates a significant difference vs. the control group; # indicates a significant difference vs. CPZ group. **p < 0.01, ****p < 0.0001, #*p* < 0.05, ##*p* < 0.01, ###*p* < 0.001, and ####*p* < 0.0001.

Motor coordination of mice was measured as the latency to fall. At the end of the demyelination stage, there was a significant difference between C and CPZ *p* = 0.0065 (Figure 3A). At the end of early remyelination stage, there was a significant difference in locomotor activity value between groups [*F*_(4.55)_ = 6.003, *p* = 0.0004]. However, there was a non-significant difference in locomotor activity between C and CPZ (*p* = 0.7446). In contrast, the comparison revealed a significant improvement in locomotor activity in mice treated with RA and IBD group compared to CPZ group (*p* = 0.0010 and *p* = 0.0196 respectively) (Figure 3B). Conversely, there was a non-significant difference shown between CPZ and mice treated with combination RA + IBD (*p* = 0.2389). At the end of late remyelination stage, there was a significant difference in locomotor activity [*F*_(4, 25)_ = 0.8271, *p* = 0.5205]. Tukey’s HSD *post hoc* test showed a non-significant difference in locomotor activity and balance between C and CPZ (*p* = 0.9001). Moreover, there was a non-significant difference between CPZ and mice treated with RA (*p* = > 0.9999), IBD (*p* = 0.9864), RA + IBD (*p* = 0.9177) (Figure 3C).

#### 3.1.3 Effects of IBD and RA and their combination on muscle strength

**Figure 4:**
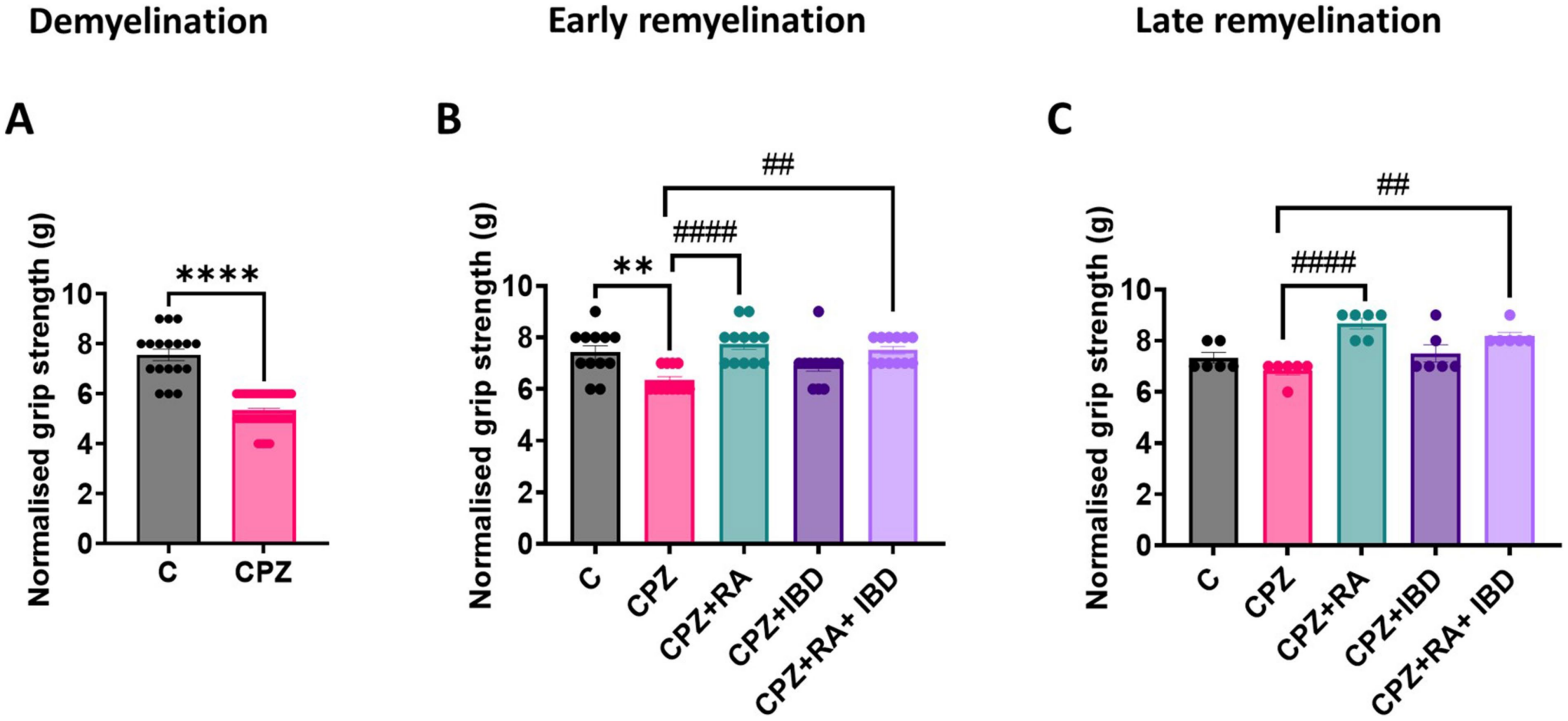
Grip strength assessment of CPZ-induced MS mice. **(A)** Grip strength at demyelination stage in CPZ-induced MS-mice, **(B)** Grip strength early remyelination. **(C)** Grip strength at late remyelination stages. At demyelination *n* = 18/C group and *n* = 54/CPZ group, early remyelination *n* = 12/group, late remyelination *n* = 6/group. * Indicates a significant difference vs. control group; # indicates a significant difference vs. CPZ group. **p < 0.01, ****p < 0.0001, ^#^*p* < 0.05, ^##^*p* < 0.01, ^###^*p* < 0.001, and ^####^*p* < 0.0001.

At the end of the demyelination stage CPZ group significantly reduced muscular strength in mice compared to the control group (*p* < 0.0001) (Figure 4A). Like the locomotor activity and motor coordination, at the end of the early remyelination stage, there was a significant difference in muscular strength value between groups [*F*_(4, 55)_ = 7.548, *p* = <0.0001]. The CPZ group was still shown a significant decrease in muscle strength compared to the control group (*p* = 0.0039). In comparison RA and the RA + IBD combination showed a significant increase in muscular strength compared to the CPZ group (*p* < 0.0001 and *p* = 0.0016 respectively). On the other hand, there was a non-significant difference between CPZ and IBD (*p* = 0.2742) (Figure 4B). At the end of the late remyelination stage, there was a significant difference in muscular strength between groups [*F*_(4, 25)_ = 9.947, *p* = <0.0001]. There was a non-significant difference in muscular strength between C and CPZ (*p* = 0.5431). While there was still a significant increase in muscular strength in RA and RA + IBD combination compared to CPZ (*p* < 0.0001 and *p* = 0.0030 respectively). Whereas the IBD is not showing any significant compared with CPZ (*p* = 0.4058) (Figure 4C).

### 3.2 Effect of IBD and RA and their combination on cognitive functions of CPZ-induced MS mice

#### 3.2.1 Effects of IBD and RA and their combination on spatial memory

**Figure 5:**
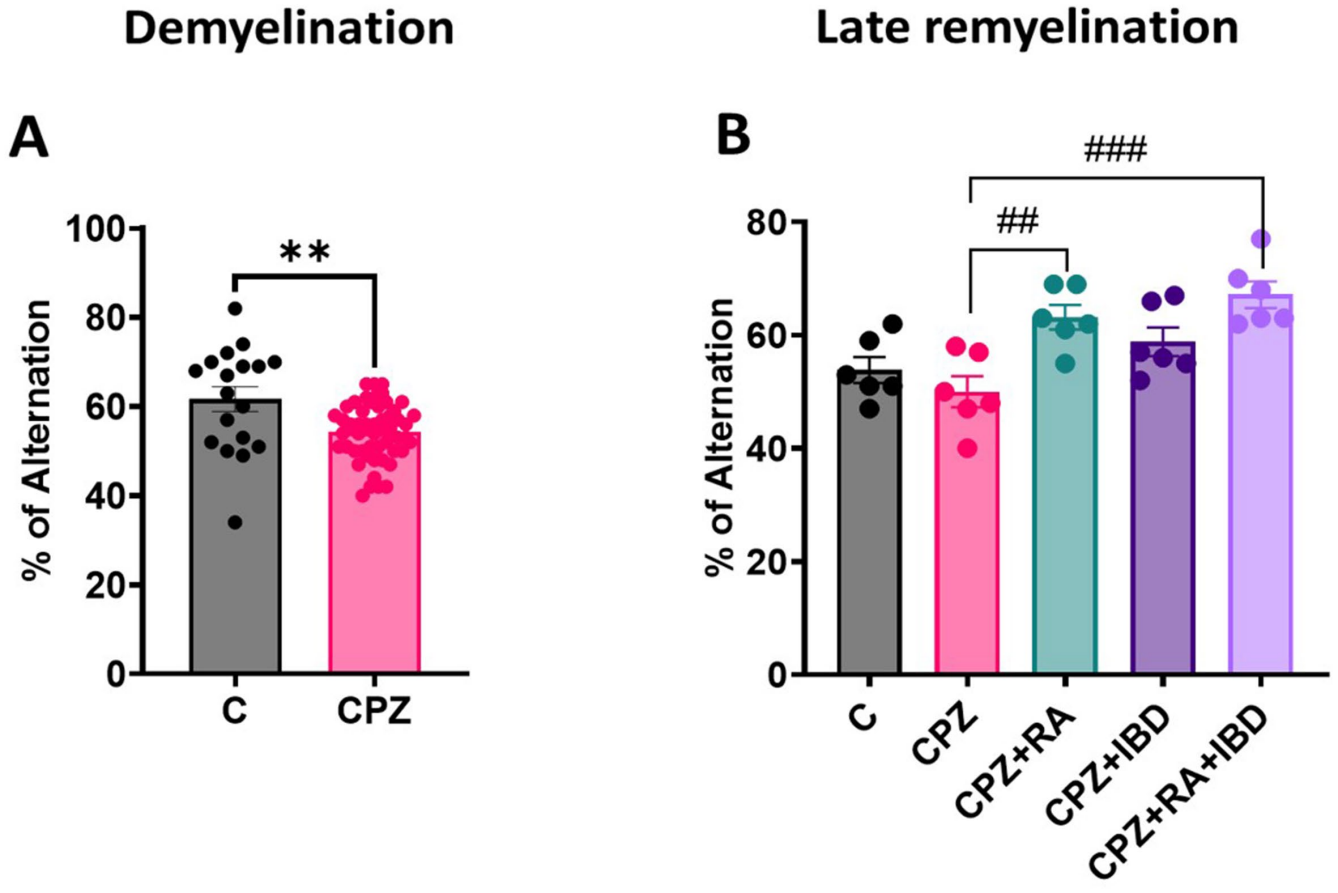
Spontaneous alternation during the Y-Maze Test: **(A)** Spontaneous alternation at demyelination stage **(B)** Spontaneous alternation at late Remyelination stage. At demyelination *n* = 18/C group and *n* = 54/CPZ group, late remyelination *n* = 6/group. * Indicates a significant difference vs. the control group; # indicates a significant difference vs. CPZ group. **p < 0.01, ^##^*p* < 0.01, and ^###^ *p* < 0.001.

At the end of the demyelination, CPZ group showed a significant decrease in the percentage of spontaneous alterations compared to the control group (*p* = 0.0011) (Figure 5A). At the end of late remyelination stage, there was a significant difference between groups in spontaneous alternation [*F*_(4, 25)_ = 8.126, *p* = 0.0002]. There was a significant improvement in spontaneous alterations in RA and RA + IBD combination compared to CPZ group which was already undergoing spontaneous remyelination (*p* = 0.0061, *p* = 0.0003 respectively). While treatment with IBD alone also enhanced spontaneous alterations, however, its effect was inferior a non-significant (*p* = 0.1056) (Figure 5B).

#### 3.2.2 Effects of IBD and RA and their combination on recognition memory and memory performance

**Figure 6:**
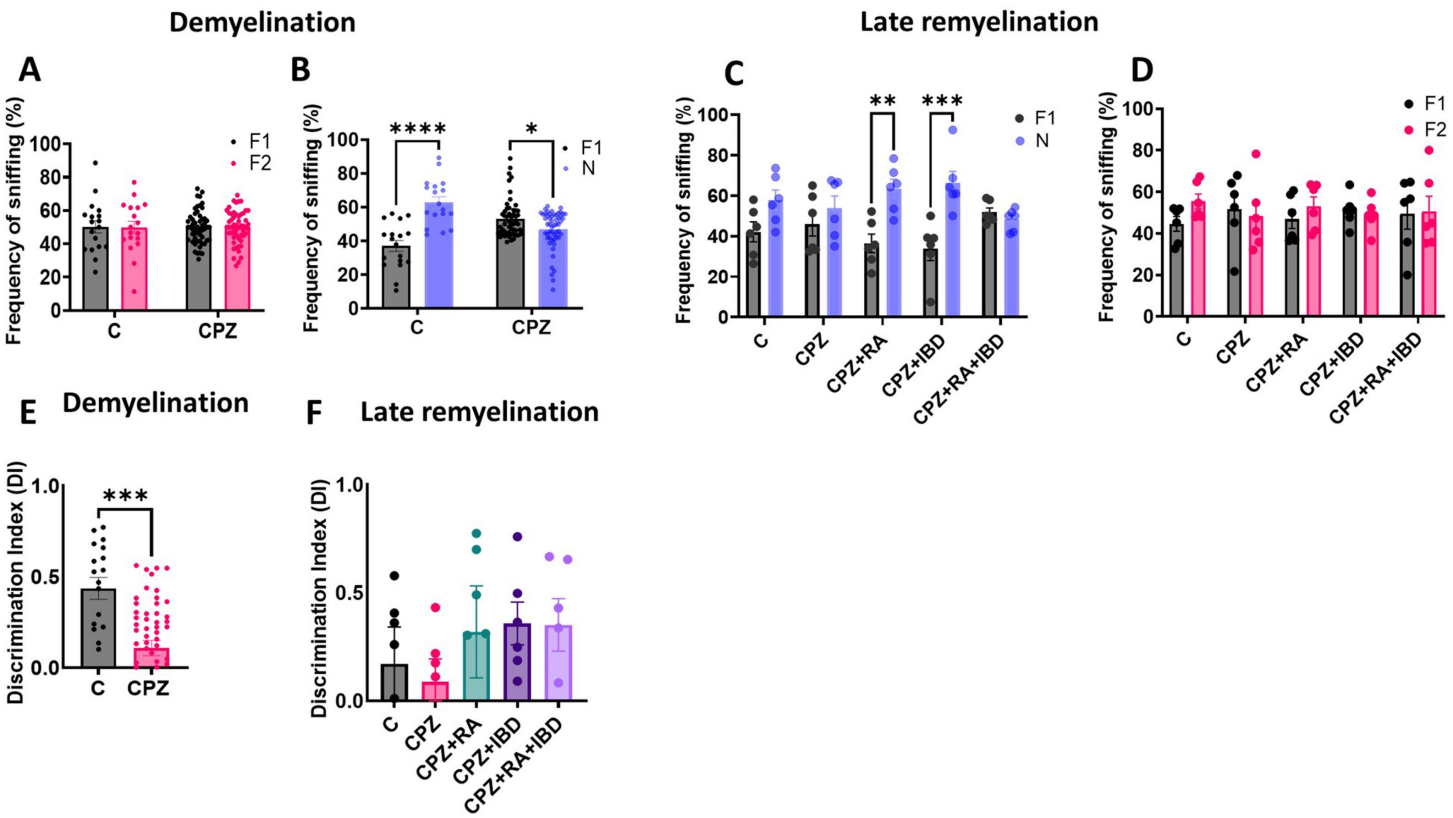
Frequency of sniffing in novel object recognition test (NORT). **(A,B)** Effect of CPZ on the percentage of sniffing in NORT at end of demyelination stage. **(C,D)** Effect of different treatments on the percentage of sniffing of CPZ-induced MS-mice at late Remyelination. A two-way ANOVA followed by Šídák’s *post hoc* test was used. **(E,F)** Discrimination index (DI) during the NORT. F1: Familiar object 1, F2: Familiar object 2, N: Novel object, Data are presented as mean ± SEM. An Unpaired *T*-test was used. At demyelination *n* = 18/C group and *n* = 54/CPZ group, late remyelination *n* = 6/group. * Indicates a significant difference, *p < 0.5, **p < 0.01, *** *p* < 0.001, ****p < 0.0001.

At the end of demyelination stage, analysis revealed no significant differences in the frequency of sniffing (%) for each familiar object within the group [*F*_(1, 140)_ = 0.01184, *p* = 0.9135]. There was no significant difference in the percentage of time spent sniffing familiar objects 1 and 2 (F1, F2) within C group (*p* = 0.9921) and within CPZ group (*p* = 0.9997) during the familiarization phase (Figure 6A). During the test phase there was a significant increase in frequency of sniffing of the novel object compared to the sniffing of familiar object within C group (*p* < 0.0001). Interestingly, a significant decrease was observed in terms of frequency of sniffing of the novel object compared to familiar object within CPZ group (*p* = 0.0156) (Figure 6B). At the end of the late remyelination stage, no significant difference was found in the frequency of sniffing (%) for each familiar object within the group during familiarization phase [*F*_(1, 10)_ = 29.22, *p* = 0.0003] (Figure 6C). However, there was a non-significant difference in frequency of sniffing of the novel object compared to the sniffing of familiar object within C group (*p* = 0.1306) and within CPZ group (*p* = 0.7747). Whereas a significant increase in frequency of sniffing of novel objects compared to familiar objects shown during test phase within RA group (*p* = 0.0013), and IBD group (*p* = 0.0001). Interestingly, the combined treatment (RA + IBD) failed to exert any significant change in terms of sniffing frequency (*p* = 0.9904) (Figure 6D).

Regarding memory performance, at the end of demyelination stage, there was a significant decrease in discrimination index (DI) in CPZ group compared to the control group (*p* = 0.0001) (Figure 6E). At the end of the late remyelination stage, the comparison showed no significant variation in DI within the group [*F*_(4, 25)_ = 0.6662, *p* = 0.6214]. The analysis revealed that there is no significant difference in DI between C and CPZ (*p* = 0.9946). Although there was a non-significant difference between CPZ and mice treated with RA (*p* = 0.8063), IBD (*p* = 0.7034), RA + IBD (*p* = 0.7215) (Figure 6F).

### 3.3 Effects of IBD and RA and their combination on the expression of MS-associated genes in CPZ-induced MS mice

#### 3.3.1 Relative expression of NFKB-p105, COX2, TNF-*α*, STAT-3 and NGF

##### 3.3.1.1 NFKB-p105

**Figure 7:**
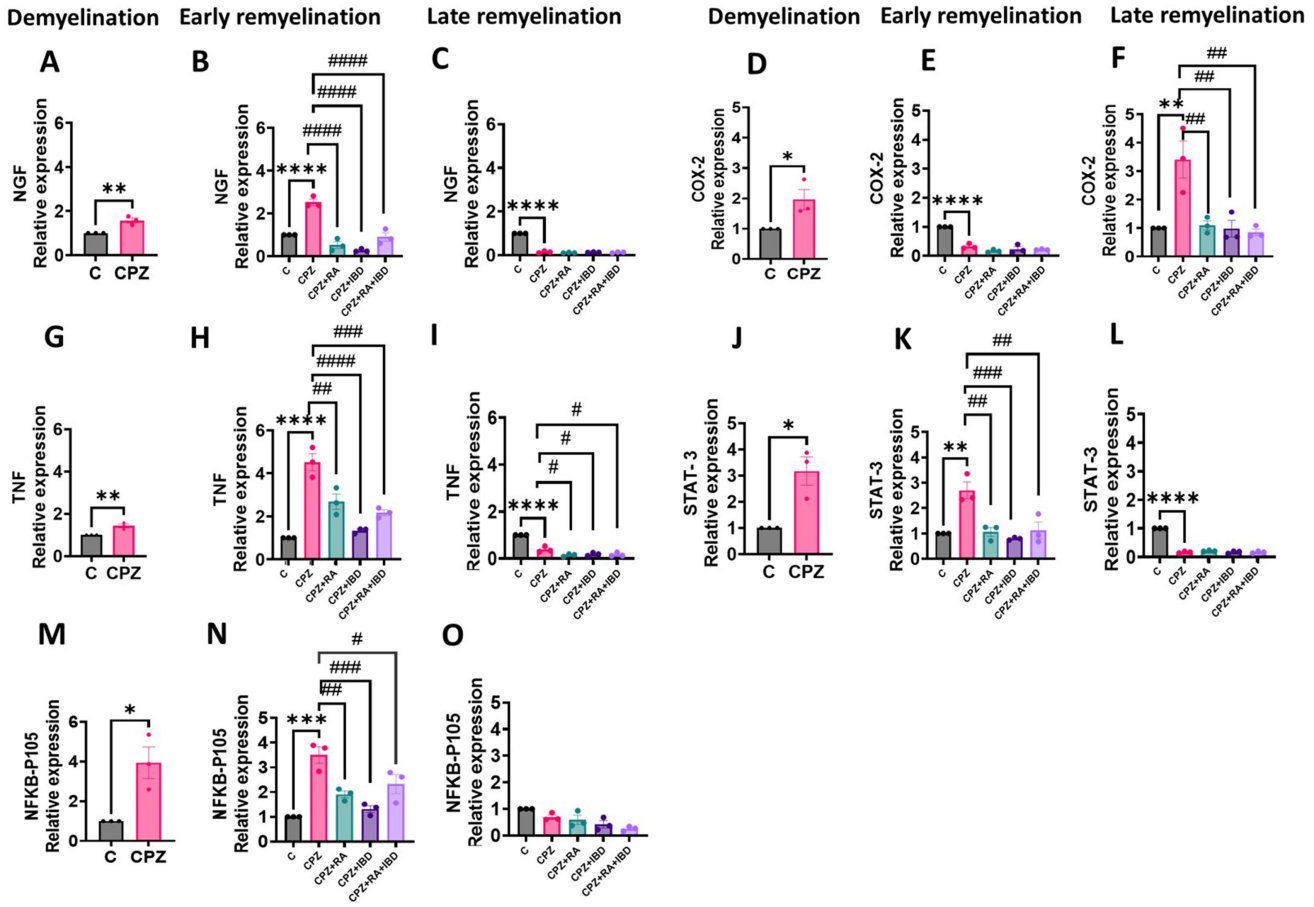
Expression levels of mRNA of MS associated genes in the brain of CPZ-induced MS mice in demyelination, and (early and late) remyelination stages. **(A–C)** NFKB-p105, (D-F) COX-2, **(G–I)** TNF-α, **(J–L)** STAT3, **(M–O)** NGF. The expression in calculated fold change relative to the expression of GAPDH reference gene. The sample size *n* = 3/group. * Indicates a significant difference vs. the control group; ^#^ indicates a significant difference vs. CPZ group. *p < 0.05, **p < 0.01, ***p < 0.001, ****p < 0.0001, ^#^*p* < 0.05, ^##^*p* < 0.01, ^###^*p* < 0.001, and ^####^*p* < 0.0001.

At the end of demyelination stage, NFKB-p105 levels were significantly increased CPZ group compared to the control group (*p* = 0.0209) (Figure 7A). At the end of the early remyelination stages, there was a significant difference in fold change of mRNA level in NFKB-p105 [*F*_(4, 10)_ = 16.11, *p* < 0.0002]. The level of fold change of mRNA in NFKB-p105 was still significantly high in the CPZ group compared to the C group in the end of early remyelination stages (*p* = 0.0040). In contrast, the comparison showed a significant decrease in mRNA fold change level in NFKB-p105 in all treated groups, including RA (*p* = 0.0038), IBD (*p* = 0.0003) and RA + IBD (*p* = 0.0257) compared to CPZ group (Figure 7B). At the end of the late remyelination stages, there was a non-significant difference in fold change of mRNA level in NFKB-p105 [*F*_(4, 10)_ = 6.874, *p* < 0.0063]. The level of fold change of mRNA in NFKB-p105 was returned to normal in the CPZ group compared to the C group (*p* = 0.2565). Although there was a non-significant difference between CPZ and mice treated with RA (*p* = 0.9444), IBD (*p* = 0.3581), RA + IBD (*p* = 0.0649) (Figure 7C).

##### 3.3.1.2 COX-2

At the end of demyelination stage, COX-2 levels were significantly increased CPZ group compared to the control group (*p* = 0.0458) (Figure 7D). At the end of the early remyelination stages, there was a significant difference in fold change of mRNA level in COX-2 [*F*_(4, 10)_ = 10.69, *p* = 0.0012]. The level of fold change of mRNA in COX-2 was still significantly higher in the CPZ group compared to the C group in the end of early remyelination stages (*p* = 0.0018). Whereas the comparison showed a significant decrease in mRNA fold change level in COX-2 in all treated groups, including RA (*p* = 0.0025), IBD (*p* = 0.0017) and RA + IBD (*p* = 0.0012) compared to CPZ group (Figure 7E). At the end of the late remyelination stages, there was a significant difference in fold change of mRNA level in COX-2 [*F*_(4, 10)_ = 56.21, *p* < 0.0001]. The level of fold change of mRNA in COX-2 was significantly decreased in the CPZ group compared to the C group (*p* < 0.0001). On the other hand, there was a non-significant difference between CPZ, and mice treated with RA (*p* = 0.1318), IBD (*p* = 0.5326), RA + IBD (*p* = 0.3976) (Figure 7F).

##### 3.3.1.3 TNF-*α*

At the end of the demyelination stage, TNF-α levels were significantly increased CPZ group compared to the control group (*p* = 0.0098) (Figure 7G). At the end of the early remyelination stages, there was a significant difference in fold change of mRNA level in TNF-α [*F*_(4, 10)_ = 30.87, *p* < 0.0001]. The level of fold change of mRNA in TNF-α was still significantly higher in the CPZ group compared to the C group in the end of early remyelination stages (*p* < 0.0001). In contrast, the comparison showed a significant decrease in mRNA fold change level in TNF-α in all treated groups, including RA (*p* = 0.0016), IBD (*p* < 0.0001) and RA + IBD (*p* = 0.0002) compared to CPZ group (Figure 7H). At the end of the late remyelination stages, there was a significant difference in fold change of mRNA level in TNF-α [*F*_(4, 10)_ = 64.93, *p* < 0.0001]. The level of fold change of mRNA in TNF-α was significantly decreased in the CPZ group compared to the C group (*p* < 0.0001). Moreover, there was still a significant decrease between CPZ, and mice treated with RA (*p* = 0.0126), IBD (*p* = 0.0389), RA + IBD (*p* = 0.0268) (Figure 7I).

##### 3.3.1.4 STAT-3

At the end of the demyelination stage, STAT-3 levels were significantly increased CPZ group compared to the control group (*p =* 0.0157) (Figure 7J). At the end of the early remyelination stages, there was a significant difference in fold change of mRNA level in STAT-3 [*F*_(4, 10)_ = 11.99, *p* = 0.0008]. The level of fold change of mRNA in STAT-3 remained significantly higher in the CPZ group compared to the C group in the end of early remyelination stages (*p* = 0.0012). On the other hand, the comparison revealed a significant reduction in mRNA fold change level in STAT-3 across all treated groups, including RA (*p* = 0.0016), IBD (*p* = 0.02939) and RA + IBD (*p* = 0.0022) compared to CPZ group (Figure 7K). At the end of the late remyelination stages, there was a significant difference in fold change of mRNA level in STAT-3 [*F*_(4, 10)_ = 519.7, *p* < 0.0001]. The level of fold change of mRNA in STAT-3 was significantly decreased in the CPZ group compared to the C group (*p* < 0.0001). Conversely, there was a non- significant difference in fold change of mRNA level in STAT-3 between CPZ and mice treated with RA (*p* = 0.3028), IBD (*p* > 0.9999), RA + IBD (*p* > 0.9999) (Figure 7L).

##### 3.3.1.5 NGF

At the end of the demyelination stage, NGF levels significantly increased in CPZ group compared to the control group (*p =* 0.0073) (Figure 7M). At the end of the early remyelination stages, there was a significant difference in fold change of mRNA level in NGF [*F*_(4, 10)_ = 42.97, *p* < 0.0001]. The level of fold change of mRNA in NGF remained significantly higher in the CPZ group compared to the C group in the end of early remyelination stages (*p* < 0.0001). In contrast, the comparison showed a significant decrease in mRNA fold change level in NGF across all treated groups, including RA (*p* < 0.0001), IBD (*p* < 0.0001) and RA + IBD (*p* < 0.0001) compared to CPZ group (Figure 7N). At the end of late remyelination stage, there was a significant difference in fold change of mRNA level in NGF [*F*_(4, 10)_ = 774.4, *p* < 0.0001]. The level of fold change of mRNA in NGF was significantly decreased in the CPZ group compared to the C group (*p* < 0.0001). Contrariwise, there was a non- significant difference in fold change of mRNA level in NGF between CPZ and mice treated with RA (*p* = 0.4105), IBD (*p* = 0.9675), RA + IBD (*p* = 0.8416) (Figure 7O).

## 4 Discussion

MS is one of the most abundant neurodegenerative diseases with an annually increasing prevalence. Until now, there has been no cure for MS, but the FDA has approved disease modifying medication that alleviates symptoms and controls progression of MS. Therefore, there is a constant need to improve the modest effect of these medications by supplementing with nutritional factors that suppress neurodegenerative and inflammatory effects of MS. In order to discover the potential role of RA as a booster for IBD. The main objective of this study was to explore the potential therapeutic effects of RA as a booster for IBD against CPZ-induced behavioral and molecular change in MS mouse model. Around 85% of patients’ diagnosis with MS showed motor function deficits such as movement, speed, balance, and 43–70% showed cognitive impairment. Thus, several behavioral tests such as open-field, rotarod, and grip strength were conducted to evaluate motor deficits, while novel object recognition and Y-maze assessments were performed to evaluate cognitive impairment in CPZ animal model. Thoroughly, our data showed that administration of CPZ for 5 weeks declined the locomotor activity, memory and muscle strength, as indicated by significant decrease in motor coordination, TDM, velocity, percent spontaneous alteration, percent sniffing and grip strength compared to the control group which is consistent with (Faizi et al., 2016; Elbaz et al., 2018; Alghamdi and Abo Taleb, 2020; Han et al., 2020; Lubrich et al., 2022; Barros and Fernandes, 2021; Omotoso et al., 2018; Jambi et al., 2024; Eo et al., 2024; Al-Otaibi et al., 2023). Furthermore, the decline in locomotor activity and memory in CPZ model is attributed to severe demyelination, activation of microglia, astrocytes, and apoptosis of oligodendrocyte in Corpus Callosum.

Neuroinflammation plays a critical role in neurodegenerative diseases where inflammatory initiate demyelination correlated to motor, learning and memory deficits. In CPZ animal model, the pro-inflammatory cytokines caused demyelination by microglia and astrocyte activation (Gudi et al., 2014; Praet et al., 2014). In the current study, we examined the relative expression levels of inflammatory genes such as (*NFKB-P105, TNF-α, STAT-3, COX-2,* and *NGF*) in mice brain during different stages of the study. The Nuclear factor kappa B subunit p105 is a component of the NFKB gene that plays a role in breaking down the myelin sheath, causing oligodendrocyte loss and axonal damage by activating astrocytes and microglia (Yue et al., 2018; Blank and Prinz, 2014). Likewise, cyclooxygenase-2 (COX-2) is an enzyme member of the oxidoreductase family that leads to inflammation and causes oligodendrocytes damage, activating astrocytes, microglia, and macrophages (Carlson et al., 2010). Moreover, Signal transducer and activator of transcription 3 (STAT-3) in activated astrocyte, microglia, and macrophage promote some of pro-inflammatory cytokines such as (TNF-α, IL-6 and NO), which leads to tissue damage and neurons deficits (Molet et al., 2016). Additionally, TNF-α is critical inflammatory cytokines that cause tissue damage (Jang et al., 2021). NGF is a polypeptide synthesized by neurons and stimulates neurons’ growth, and function (Pouso and Cairrao, 2022). The results showed a significant increase in expression of NFKB-p105, COX-2, TNF-α, NGF, and STAT-3 in CPZ mouse brain compared with control group which is consistent with previous studies (Elbaz et al., 2018; Ghaiad et al., 2017).

At the early remyelination stage, our results showed that the locomotor activity and muscle strength of the CPZ group was significantly decreased compared to the control group except for rotarod performance, which was improved as in control group (Han et al., 2020; Al-Otaibi et al., 2023; Abo Taleb and Alghamdi, 2020). These behavioral defects were accompanied with elevated levels of (NFKB-p105, TNF-α, STAT-3, COX-2, and NGF), which agrees with previous findings (Elbaz et al., 2018; Ghaiad et al., 2017). On the other hand, all these results ameliorated and became equivalent to the control group in the late remyelination stage except for rotarod performance which remained unchanged while the expression levels of *COX-2, TNF-α, NGF, and STAT-3* genes showed a significant decrease compared to control group.

Regarding the effect of RA, IBD, and their combination at remyelination stages, our results showed that the administration of RA alone led to significant improvements in various behavioral performances compared to CPZ group, as evidenced by enhanced motor coordination, velocity, TDM, and recognition memory. The improvement observed in motor coordination can be attributed to several key factors. RA’s well-known antioxidative and anti-inflammatory properties help reduce oxidative stress, prevent neuronal damage, and alleviate inflammation, which can otherwise impede motor coordination and balance (Ahlemeyer et al., 2001; Kang et al., 2024). Additionally, studies suggest that RA can modulate synaptic strength and connectivity, which plays a crucial role in synaptic plasticity—a fundamental process for motor learning and fine-tuning motor skills (Hsu et al., 2019; Rothwell et al., 2017).

The improvement in velocity and TDM reflects the broader positive effects of RA. It has been shown to reduce microglial activity (Pouso and Cairrao, 2022), improve the maturation of oligodendrocytes (Morrison et al., 2020), and prevent axonal damage, all of which are critical for maintaining efficient neural function. Furthermore, RA has been found to promote muscular strength, particularly during the early stages of remyelination, compared to the CPZ group.

The improvement in cognitive function seen after RA administration can be attributed to several factors. RA has been found to promote neurogenesis in certain brain regions, such as the hippocampus (Bonnet et al., 2008), which may enhance learning, memory, and cognitive flexibility (Behairi et al., 2016; Pallet and Touyarot, 2015). Additionally, RA can modulate neurotransmitter systems in the brain, including the glutamatergic and cholinergic systems (Mufson et al., 2008), both of which are crucial for cognitive functions and synaptic transmission. These factors provide potential explanations for the observed improvements, suggesting they may contribute to the enhanced spontaneous alternation in mice following RA treatment.

The results observed with RA in the novel object recognition test may be attributed to the activation of RA receptors expressed in brain regions like the hippocampus. This activation of retinoic acid receptors (RARs) has been shown to enhance memory formation (Bonhomme et al., 2014; Sharma et al., 2022). These factors contribute to the enhanced behavioral performance observed in mice treated with RA. To the best of our knowledge, no previous studies reported the impact of RA administration on behavioral tasks in CPZ model.

Moreover, IBD administration alone led to significant improvements in various behavioral performances compared to CPZ group, as evidenced by enhanced motor coordination, and recognition memory at late remyelination. The improvement in coordination can be attributed to various properties of IBD including reducing inflammation that disrupts neuronal signaling in the CNS and modulating of neurotransmitters (Ray et al., 2014). The enhancement seen in the recognition memory deficits at late remyelination it might be due to reducing inflammation and beta-amyloid deposition (Kadota et al., 2022; Oliveros et al., 2023).

However, despite showing a significant result in the novel object exploration test, there was no significant difference in the discrimination index compared to the CPZ group. These findings suggest that while overall exploratory behavior may vary between groups, the ability to distinguish between familiar and novel objects remains similar across all groups. It is important to note that the small sample size may limit the generalizability of these results and warrant further investigation with larger sample sizes. In addition, we found that combined treatment with RA + IBD also improved muscular strength and recognition memory in Grip Strength and Y-maze tests at late remyelination.

To prove the positive impacts of RA and IBD (alone and in combination) on behavior defects and remyelination rate, the expression levels of NFKB-P105, TNF-*α*, STAT-3, COX-2, and NGF were conducted. Our results showed that treatment with RA significantly decreased the expression levels NFKB-P105, TNF-α, STAT-3, COX-2, and NGF in mouse brain at end of early remyelination stage compared with the CPZ group. The explanation of RA result might be attributed to their anti-inflammatory and antioxidant effects (Siddikuzzaman, 2013). Several studies in AD animal model were showed the upregulation in expression of retinoic acid receptor (RAR)-b and transforming growth factor-b1, which directly decrease the nuclear factor-jB (NFjB) transcription activity; consequently, TNF-α and NO activity were inhibited (Tjalkens et al., 2017). Both the IBD and RA + IBD groups showed comparable results to the RA group. The explanation of IBD result might be attributed to their anti-inflammatory and antioxidant effects (Baudouin et al., 2021), whereas studies showed IBD suppressed the activity of NO, ROS, IL-1β, IL-6 and TNF-α, and enhanced the IL-10 and NGF (Mizuno et al., 2004; Fox et al., 2016).

The current study reveals new discoveries about the anti-inflammatory impacts of RA, IBD, and RA + IBD in the CPZ model that have not been documented before. It is worth mentioning that RA and IBD are known anti-inflammatory drug that has been thoroughly researched for their impact on various inflammatory conditions. However, this research does not indicate any interaction between RA and IBD, as there was no significant effect of RA on the action of IBD. While Both RA and IBD were administered independently, and their effects appeared to be separate rather than combined. More research is necessary to clarify the specific mechanism by which the effect of RA and IBD administration influences behavioral and molecular changes in the CPZ model.

## Data Availability

The original contributions presented in the study are included in the article/supplementary material, further inquiries can be directed to the corresponding author(s).
