# Supplementary material for: Potential therapeutic effects of ibudilast and retinoic acid against cuprizone-induced behavioral and biochemical changes in mouse brain
Source: Front Mol Neurosci. 2025 May 20;18:1567226. doi: 10.3389/fnmol.2025.1567226 (PMC12129909; doi:10.3389/fnmol.2025.1567226)
Supplement: Supplementary file 1 [file Table_1.docx]

Table S1: List of primers used for quantitative real-time PCR

| **Target gene** | **Sequence (5'->3')** |
| --- | --- |
| **COX-2 Forward** | TGAGTACCGCAAACGCTTCT |
| **COX-2 Reverse** | CAGCCATTTCCTTCTCTCCTGT |
| **TNF Forward** | GGTCCCCAAAGGGATGAGAAGT |
| **TNF Reverse** | TTGCTACGACGTGGGCTAC |
| **NFKB-P105 Forward** | ATGGCAGACGATGATCCCTAC |
| **NFKB-P105 Reverse** | TGTTGACAGTGGTATTTCTGGTG |
| **NGF Forward** | ACTGGACTAAACTTCAGCATTCC |
| **NGF Reverse** | GGGCAGCTATTGGTGCAGTA |
| **STAT-3 Forward** | CAATACCATTGACCTGCCGAT |
| **STAT-3 Reverse** | GAGCGACTCAAACTGCCCT |
| **NRF Forward** | TGTAGATGACCATGAGTCGCTTG |
| **NRF Reverse** | TATTGAGGGACTGGGCCTGAT |
